# The Use of Jaborandi

**Published:** 1907-05-11

**Authors:** 


### Points in Treatment.

### THE USE OF JABORANDI.

Jaborandi in the crude form consists of the dried leaflets of a rutaceous shrub, imported from various places in South America, principally from Pernambuco, in Brazil. The original shrub from which the most active preparations used to be made was the *Pilocarpus Jaborandi*, but unfortunately this particular variety of the plant is now extremely scarce, and other kinds are used for supplying the crude Jaborandi which is exported to England.

The questions arise: Are these other kinds of leaves of any use medicinally? If so, which kinds are the most useful? We will answer these questions presently.

It is well known how valuable a drug pilocarpine is. As a rule it is administered hypodermically, in the form of a solution of one of its salts. Pilocarpine itself is a colourless syrupy liquid, which has been prepared synthetically from pyridine, *viâ* pilocarpidine. Its salts are mostly crystalline, the nitrate being the officinal preparation with a dose of $\frac{1}{20}$ to $\frac{1}{2}$ grain; the other salts that are used, though unofficinal, are the hydrochloride ($\frac{1}{20}$ to $\frac{1}{3}$ grain), the salicylate ($\frac{1}{20}$ to $\frac{1}{2}$ grain), and the carbolate, of which a dose of 3 to 5 c.c. of a 1 in 5,000 solution is sometimes used hypodermically in phthisis and malaria. The value of pilocarpine nitrate as a diaphoretic is well known, and it is commonly employed in eclampsia, severe nephritis, and allied conditions; it is also of use in diminishing the urine in some cases of diabetes.

If pilocarpine is so useful, why should not jaborandi preparations do good too? They would have the advantage of being given by the mouth instead of hypodermically; and yet there is an almost unanimous opinion that tincture of jaborandi, for example, is almost useless in comparison with pilocarpine nitrate. This would not be of much importance if the drug were only used as an emergency measure, as it is in uræmia; but if the jaborandi were really serviceable, there can be little doubt that it would be a great boon in the long continued treatment of nephritis, diabetes, and possibly other conditions in which the continued administration of pilocarpine hypodermically is almost out of the question. One other condition in which jaborandi, if active, is said to have a very beneficial effect is in the treatment of alopecia by local application to the scalp.

It might be argued that jaborandi does not produce the same effects as pilocarpine because, given by the mouth, some of the pilocarpine it may contain is likely to be destroyed by the gastric juice; this argument would not apply, however, in cases where the drug is given per rectum; and yet even per rectum, neither of the officinal preparations of jaborandi (extractum jaborandi liquidum, dose 5 to 15 minims, and tinctura jaborandi, dose $\frac{1}{2}$ to 1 fluid drachm) appears to produce more effect than when given by the mouth. Another, and an exceedingly important, reason why jaborandi preparations are comparatively inert, is that the great bulk of commercial jaborandi contains practically no alkaloid at all. As we have mentioned already, all sorts of leaves are sent over to England labelled jaborandi; some are, for example, *Pilocarpus pennatifolius*, *Pilocarpus microphyllus*, *Pilocarpus trachylophus*, *Pilocarpus Selloanus* from Rio de Janiero, and several varieties of Brazilian *Piper*, which are sold as jaborandi leaves. *Pilocarpus Jaborandi* is now so rare that much of what is exported as such is worthless, yielding no alkaloid at all upon analysis. The *Pipers* are equally unserviceable. The leaves of *Pilocarpus pennatifolius* yield very small quantities of pilocarpine, varying from 0.07 to 0.15 per cent.; of all the varieties upon the market the two which contain most pilocarpine are *Pilocarpus microphyllus*, whose leaves yield from 0.55 to 0.63 per cent. of the alkaloid, and *Pilocarpus trachylophus* (Ceara Jaborandi), giving 0.75 per cent.

It is very important that the source of the jaborandi should be known when it is prescribed; it is more than likely that much of the disfavour into which tincture of jaborandi has fallen is due to the absence of the alkaloid from the preparation used. The tincture is not standardised; the druggist makes it of the strength of 1 part of the dried leaves in 5 parts of 45 per cent. alcohol, and without analysis there is no guarantee of the activity of the sample. It is quite obvious that the medical man would be glad to know the percentage of alkaloid in the sample of the tincture he is buying; but, failing this, the next best thing is for him to make certain that the tincture has been made from *Pilocarpus microphyllus* leaves, or, better still, those of *Pilocarpus trachylophus*, and not from any of the other varieties of jaborandi, which are relatively or absolutely inert.

The pilocarpine which we use is by no means always pure; this may account for its lack of action in some severe cases of nephritis where diaphoresis is urgently indicated. It is not quite easy to distinguish pilocarpidine from pilocarpine nitrate, and some commercial samples of pilocarpine nitrate contain as much as 50 per cent. of pilocarpidine. The dose of such a sample would need to be doubled if a given pilocarpine effect were required, whilst at the same time the pilocarpidine might be producing undesirable effects. The relation between pilocarpidine and pilocarpine is shown by the fact that the former is a stage in the synthesis of the latter from pyridine. How, then, can the medical man be sure that the drug is fairly pure? He should ask its *melting-point*; the melting-point of pilocarpine nitrate is 176-178° C.; that of pilocarpidine nitrate is 158° C. Some samples of so-called pilocarpine as found upon the market melt at 158° C., and probably contain hardly any pilocarpine at all; the medical man should refuse to buy any pilocarpine nitrate whose melting-point is less than 170° C., and he should try to get it with a melting-point of 178° C.

